# Five Qualitative Research Concepts Grounded in Anthropological Methods for Teaching Design in Healthcare

**DOI:** 10.3390/healthcare10020360

**Published:** 2022-02-12

**Authors:** Constanza Miranda, Julián Goñi, Nicole Labruto

**Affiliations:** 1 Department of Biomedical Engineering, Johns Hopkins University, Baltimore, MD 21218, USA; 2 DILAB School of Engineering, Pontificia Universidad Católica de Chile, Santiago 7820436, Chile; jvgoni@uc.cl; 3 Department of Anthropology, Johns Hopkins University, Baltimore, MD 21218, USA; nlabruto@jhu.edu

**Keywords:** anthro-design, qualitative research, health design education, ethics, design-based research

## Abstract

Biomedical engineering, engineering, and design in health programs around the world have involved human-centered design as part of their undergraduate curriculum. The disparities evidenced during the COVID-19 pandemic and the rapid developments of biotech startups have highlighted the importance of preparing professionals in the health areas for undertaking rigorous, empathetic, and ethical research. In addition to working with human-driven information, students in the health areas are challenged to deal with technical developments that involve legal and ethical concerns deeply rooted in sociopolitical issues and human rights. Concerned with how to achieve a better understanding of behavior in designing for healthcare, this article describes the rationale behind teaching qualitative research in healthcare for biomedical engineering and engineering design education. Through portraying different healthcare designs resulting from an engineering design course, it describes the instruction of qualitative-driven concepts taught to biomedical engineering, design, and premed undergraduate students. Using a design-based research approach, we look to increase the chances of adoption of the presented qualitative research concepts in educational design in health programs. We deliver five tested research tools that better prepare students to carry out more rigorous, respectful, and aware qualitative research in health areas for the development of novel solutions.

## 1. Introduction

### 1.1. The Social Turn in Design Education for Healthcare

Today, a new paradigm for health education is on the rise. Through core curricular courses such as design teams courses [1], biomedical engineering (BME) undergraduates are asked to resolve issues prompted by medical professionals [1,2,3]. In the case of engineering capstone courses, a substantial number of the projects developed by students are from the medical area [4]. Programs that mix design and healthcare are becoming more and more prolific in the United States (i.e., the Design for Health program at the University of Texas at Austin [5], Rice University’s Bioengineering in Global Medical Education [6], Stanford University’s Biodesign [7], Duke University’s BME Design Fellows [8], Johns Hopkins University’s Master in Bioengineering, Innovation and Design [9], among others). Through the years the health topics presented have become increasingly social in nature, requiring students to be more aware of human involvement in the adoption of new technologies. Some of the projects pitched to our undergraduate students in the last years are related to patient compliance, aging and wellness, and preventive medicine. All of these imply not only creating a novel solution but understanding thoroughly the human and social aspects of design.

With the pandemic crisis of COVID-19 and the first rollouts in vaccination, strong inequalities in the distribution and access to healthcare have been acknowledged by the general public [10,11]. In addition, the Food and Drug Administration (FDA) in the US has provided regulatory frameworks for artificial intelligence (AI) and machine learning developments in the health industry [12]. This follows up their proposed regulatory discussion paper published in 2019 [13]. In this complex environment, tech companies and their engineers have been scrutinized for the way they deal with data, not only in terms of privacy and transparency [14], but also for how they impact people’s subjectivity. For instance, increased connectivity can also lead to subjective isolation and mental health issues [15]. Lauer [16] suggests that harm to mental health is not incidental but is part of a technological advancement process that has focused on achieving successful business outcomes without regard for the mental health hazards users face [16]. These issues highlight the importance of ethics in AI [9] and in design for healthcare more broadly.

Humanistic approaches that depict the intricacies of race in healthcare [17,18] and reproductive disparities [19,20] have been more present in education through formal medical humanities course concentrations. Nonetheless, these are not mandatory courses for health science students. For students in BME, these provide “optional” coursework. In the case of engineering, science, technology, and society (STS) programs in universities such as Virginia Tech, MIT, and the University of Maryland, or anthropology and design courses such as the ones at Olin College or Pontificia Universidad Católica in Chile [21] have looked to bridge the gap between engineering, the sciences, and the humanities. 

In engineering sciences, curricula that have traditionally focused on the acquisition of technical and scientific skills in the healthcare field [22], there is a misalignment between the educational methods used to impart social competencies and the social turn that has been identified in engineering education [21,23]. By social turn we mean a turning point in engineering education where the application of social science and humanities principles becomes a central aspect of engineering practices [21,23]. In this sense, this social turn in engineering education highlights the social impact of engineering practices and calls for more critical, responsible, and culturally aware educational practices [21]. As a subset of engineering, biomedical engineering and other health design programs should include education in critical humanistic perspectives, such as those used in initiatives that focus on patient-centered medicine [24] and narrative medicine [25,26,27]

### 1.2. Designing for Healthcare, a Qualitative Research Endeavor

Designing technological developments for the health sector has been a key element in biomedical engineering education. As Petroski has stated, design is the “soul of engineering” [28]. As a subdiscipline of engineering, a major component of educating BME students is to have them solve ill-defined and open-ended issues [29] using a design process. According to the Accreditation Board of Engineering Technology (ABET) this engineering design process must be iterative, work within constraints, evaluate technical performance, and provide user validation among other aspects [30]. Technical and technological creations in healthcare affect and are shaped by the lives of real people, value systems, and ways of life. This constitutive social dimension requires that engineers possess the ability to conduct innovation and development in responsible, reflexive ways [31]. These ever-present social elements challenge students to deal with imperfect data and consider strategies derived from the humanities and the social sciences.

**Figure 1:**
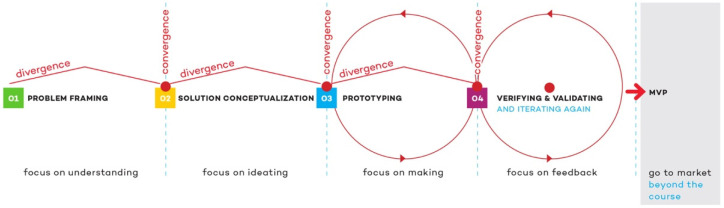
The process taught to undergraduate design teams in a leading BME undergraduate design program. The first phase relies heavily on qualitative research or what has been called “needs finding” in similar programs.

Most of the courses that intend to address open-ended clinical problems in biomedical engineering use the design thinking process, which emphasizes divergent and convergent thinking processes, and which locates an abstracted ill-defined user at the center of experience. Although popularized by IDEO in the 1990s, it was in 1965 that Newell had written about this form of creative divergent-convergent thinking process in a scientific publication [32]. Our analysis of programs in engineering design, biomedical engineering design, and design for health has shown that they do not differ much from each other. They generally involve an individual-centered process that uses divergent and convergent iterative thinking to achieve a solution for a specific context. Figure 1 shows the standard design process that undergraduate students in a leading US-based BME program underwent in 2021. It is similar to other design or engineering design processes taught in engineering and design schools, and it is very similar to the one undertaken at Pontificia Universidad Católica de Chile as published in Miranda et al. [33] and that will be revisited in this article.

Other programs such as Stanford’s Biodesign executive education program [7] and Johns Hopkins’s Center for Biomedical Innovation and Design (CBID) master’s program [9] use a slightly modified human-centered approach in their design process. This process has followed what Stanford’s D-School has called “need finding” in their curriculum [34]. Need finding is an applied and succinct way of doing qualitative assessments in the field. It mostly relies on open-ended interviews, shadowing, and other sorts of short observations that can inform the engineering designer about what is going on [28]. These processes are not so different from what design educators have seen in human-computer interaction (HCI) design. HCI uses “goal-directed design” [35,36,37] to eschew purely economic models [38] in favor of creating a more memorable user experience. Some may argue that this user-centric model is enough to fulfill the social requirements and responsibilities of developing new technologies for the health sector. However, is this type of emphasis on user experience of new technologies sufficient in fulfilling the responsibilities of a health technology sector that has systematically failed to consider social aspects of design in historically and culturally informed ways? Furthermore, the human-centered design process is bound to the demands of business models, intellectual property regimes, and FDA regulations in healthcare [39]. Though the field and curricula in healthcare design have taken important steps toward placing users at the center of their design process, we contend that this model is not sufficient to achieve detailed, equitable representations of individuals and communities and the multivocal ethics [39] required for participatory engagement in healthcare design.

### 1.3. Anthropological Methods as a Framework for Qualitative Assessment

Since the 1950s (and even before), design has been interested in so-called human factors [40]. Works such as Henry Dreyfuss’s The Measure of Man [41] and Designing for People [42] inaugurated a paradigm shift in design that centered on the importance of users in designers’ considerations. However, a crucial shortcoming of the standard design process is the lack of opportunity for the designer to contextualize “the user” as a socially embedded, multidimensional person, beyond a unidimensional archetype [21]. In the 2000s [43] designers began adopting tools borrowed from ethnography to attempt to achieve a more ethical design praxis [39]. They sought to employ rigorous methods used in the social sciences to understand people’s motivations in healthcare settings [44]. However, borrowing ethnographic methods is not sufficient if it does not also include the adoption of the ethical and epistemological frameworks that undergird the use of these tools in analyzing social experience, as is done within the field of anthropology. What anthropology, at least since the postmodern debates of the 1980s [45,46,47], offers is a research program that insists on the radical parity of other forms of knowledge with that of the ethnographer. Understanding this sensibility requires sustained education on the structural economic, racial, gendered, and ableist disparities that inform contemporary social life, beyond an end goal of designing health technologies. Utilizing both the methods and critically informed orientation of anthropological research affords a profound understanding of the complexity of social contexts. Designing for healthcare requires more social responsibility and more engagement with communities in codesigning solutions. This move toward understanding both how structural inequalities are lived and how lived experience is structured demands a critical and broad revision of design practices [40].

In anthropological research, the primary strategy used to gain an understanding of social activities and expressions in particular contexts is ethnography. This is not only a tool for research but a way to understand knowledges and practices [41]. The application of anthropological frameworks in BME programs would allow students to collect better-informed field research data and to make sense of it beyond technical analysis. A study published in 2021 found that engineering students who went through anthro-design training experienced significant changes in their epistemic beliefs [21]. Analyzing qualitative data through an anthropological theoretical framework proved to be key in encouraging more empathetic, humane, and self-aware representation in engineering design [21]. These results are promising, and they present robust evidence supporting the instruction of qualitative research methods in BME and design for health.

While extensive, long-term ethnographic immersion is necessary both to train anthropologists and to produce anthropological data, the pedagogical intervention we suggest here does not aim to contribute to the field of anthropology per se. Instead, we seek to augment human-centered design practices that employ isolated, cursory ethnographic methods while excluding the political, historical, and ethical contexts within which such data is generated and collected. We point to the resounding benefits of utilizing the robustness of anthropology as a methodological lens with which to understand the vastness of human experience and social relations, as opposed to a purely technical focus with little consideration of broader social connections. To that end, we advocate for richer epistemological engagement in biomedical design such that codesign becomes a necessary aspect of responsible, reflexive innovation in the health field.

As data showed in the article “Embracing the Social Turn: Epistemic Change in Engineering Students Enrolled in an Anthro-design Course” [21], the use of anthropology as an epistemic framework can encourage students to learn from and work with individuals from other social contexts in creating design solutions. We see a unique opportunity to build educational scaffolding for pupils by training them to recognize and elevate alternative ways of knowing and being through more ethically engaged, cross-disciplinary interventions [21].

## 2. Materials and Methods

### 2.1. A Design-Based Research Approach

For this article, we used a design-based research (DBR) approach to showcase in a descriptive way the results of working with qualitative research tools informed by anthropological theory in design health projects with undergraduate students. DBR is an applied research methodology aimed at improving educational practices through mixed methods and interdisciplinarity [48]. As Wang & Hannafin define it, DBR is “a systematic but flexible methodology aimed to improve educational practices through iterative analysis, design, development, and implementation, based on collaboration among researchers and practitioners in real-world settings, and leading to contextually-sensitive design principles and theories” [49]. DBR is not a sequence of predefined steps, but rather a collection of design and research actions aimed at studying and improving learning spaces in their original contexts [50].

In this sense, DBR looks to augment the transfer and translation of conventional educational strategies for an improved practice [51]. The practical nature of DBR classifies it as applied research, yet its role in producing generalizable theories and insights also qualifies it as theoretical research [51]. The core of this strategy is iteration, and we are using it in several programs to embrace continuous improvement.

### 2.2. An Engineering-Design Program as Context for This Research

We have applied this design-based research to the engineering design program at Pontificia Universidad Católica de Chile through the major in engineering, design and innovation. In its eight-year existence, this undergraduate program at the School of Engineering has involved anthropological qualitative research methods within its core curriculum through an intensive semester-long anthro-design course, which includes applied ethnographic immersion for its duration. The semester-long, 10 h per week course involves around 45 students ranging from 19 to 22 years old. Instructors have backgrounds in the humanities and social sciences. Teaching assistants are advanced engineering students who have previously excelled in the course. Following this research-intensive anthro-design course, the same students take a design course. There they use the knowledge they have gained to work on designing a product/device with an external counterpart. Every year, students’ teaching evaluations, feedback from the teaching assistants, and internal studies published by the institution [52,53] have facilitated improvements to the content and lectures of the course. The following information is built on the results from an epistemic change assessment published recently in the *International Journal of Technology and Design Education* [21]. The results from this effort evidence the way students changed their approach to design and qualitative research thanks to the anthropological concepts and frameworks learned in the course. The outcomes of their designs for the health sector, described in the results section of this article, evidence long-term learning in the application of their qualitative research skills. This article will showcase in a descriptive way examples of the resulting designs completed for the medical/ healthcare sector in the design studio.

### 2.3. Teaching an Interactive Qualitative Research Process

**Figure 2:**
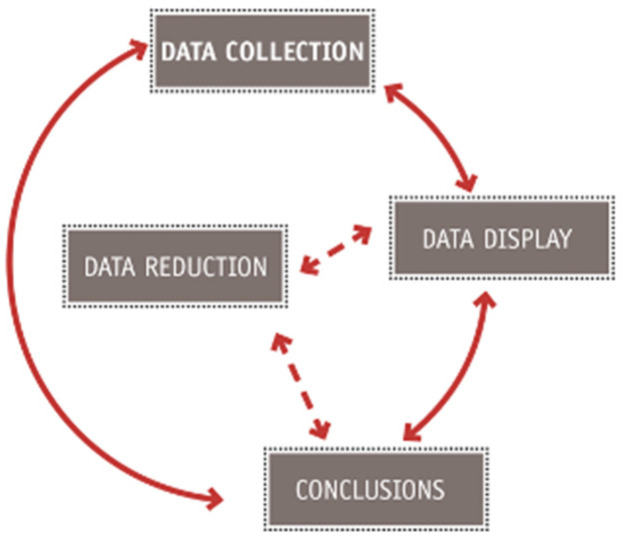
Visual adaptation of Miles and Huberman’s interactive research model.

In the anthro-design course, the qualitative research process in Figure 2 is presented to students. It is based on the interactive research model published by Miles and Huberman [54]. Instruction happens in the form of lectures and in-class activities to cover the content of the theoretical claims. This model implies that research is not static. It is provided under a constructivist paradigm that presumes that reality is cocreated among social actors, including the participants in the design process. 

### 2.4. Instructing Applied Qualitative Research Grounded in Anthropological Theory

For the initial part of the course, students are required to “understand” or “immerse” themselves in an issue prompted by a clinician or someone in the medical sector. Embarking in an interpretive act of qualitative inquiry is not easy for individuals such as engineers, biomedical engineers, or biomedical scientists who have been trained under a positivist paradigm. We introduce the concepts of culture and ethnography to understand and describe social action. To cross the disciplinary boundaries and begin to think anthropologically, we touch upon the content of foundational authors such as Clifford and Marcus [46], Geertz [55,56], Wolcott [57,58,59,60], Lassiter [61,62], Schensul and LeCompte [63], Schensul and Schensul [64] and more contemporary ones such as Bernard [65,66], Guest and Namey [67], Pink [68,69], Beebe [70] among others. This grounding sets the stage to link anthropology with technology development in the health sciences. Particularly, the ethnographic work of Pearl Katz [71] in “The Scalpel’s Edge: The Culture of Surgeons” provides a good example of what a “thick description” [56] of culture can offer to developing solutions in healthcare.

### 2.5. Instructing Five Basic Qualitative Research Concepts

Apart from instructing the anthropological framework and how it resonates with technological developments in healthcare, engineering, and technology, we have determined at least five qualitative research techniques or concepts that are important to instruct undergraduate students. As opposed to the way empathy is instructed in design thinking frameworks, these tools are grounded in anthropological theory. There is a vast amount of literature available on the theorization, application, and failures of each one of them. In addition, they are usually observed by professional institutions such as the American Anthropological Association which now and then provides guidance to their application in the field. The following description provides an overview of what it is taught in the classroom.

#### 2.5.1. Informed Consent

In addition to the students’ training on human subject research imparted by the IRB (Institutional Review Board) or ethics committees, students need to be trained in how to do an informed consent [72]. From praxis, we know there is a wrong perception that if the data is not going to be published, having written informed consent does not matter. Informed consent matters because it acknowledges the power dynamics in any type of research endeavor, even in an applied setting. Informed consent is often viewed as the central piece of the subjects’ protection. The objective is to ensure that people understand what it means to participate in the research and let them know that they can decide freely whether to engage in the research or not. In the case of participant observation techniques, it is important that there is consent from the people in participating and also regarding the dissemination of the data in ways that are not endangering their integrity [73]. Particularly, we talk about the informed consent guidelines provided by the American Anthropological Association since it discusses the importance of the quality of the consent and not only the format [74].

#### 2.5.2. Fieldnotes

Taking fieldnotes is part of the core anthropological training for the field. Fieldnotes are the way to record and articulate what the researcher is witnessing in an ethnographic endeavor. The objective of this data collection is to add detail for ultimately creating a “thick description” [56] of the events. It is important to understand that fieldnotes are a representation of “self”, and a symbol of personal identity [75] in regard to the researcher as an instrument of data collection. We talk about types of fieldnotes such as field jotting, the log, and the researcher’s diary. We review examples from Emerson, Fretz, and Shaw’s “Writing Fieldnotes” [76] on descriptive, analytical, and methodological fieldnotes. We discuss the experiences of past students and ourselves as instructors conducting fieldwork. In healthcare research, fieldnotes are relevant due to the impossibility of using recording devices in operating rooms or with patients. This form of textual data collection can be collected and triangulated with other more visual sources or data recording.

#### 2.5.3. Observation 

Nonparticipant and participant observation are key in ethnographic research. We usually instruct using nonparticipant observation for the initial stages of immersion in the field. Observation allows us to explore a certain topic or behavior without necessarily interacting with the participant. There is no involvement of the researcher in the observed scene. Nonparticipant observation can help identify behavioral patterns, common journeys, and repetitive interactions. The risk is that our research can be taken over by our own mental models or biases. This is where participant observation is key. Participant observation looks to achieve immersion but is unrefined. One goes, observes, and asks. On the other hand, observation can be useful for controlled environments, testing products, or carrying out quasi-experiments. 

#### 2.5.4. Open-Ended Interviews

We try to incorporate different points of view and data sources to enhance qualitative research validity in its own context [77]. This can be done through researcher triangulation, theoretical triangulation, or methodological triangulation. Particularly, we instruct the students to contrast their observational data with information coming from interviews. Interviews allow the researcher to understand observed events from the participant’s point of view. The researcher can get details on what’s going on and on the feelings and perceptions of the participants involved. Depending on the level of control that the interviewer has, they can be informal, unstructured, semi structured, or structured [65]. Particularly, for the initial phases of the design process in healthcare, we teach more unstructured and semi structured interviews. These are more centered on discovering and understanding the problem from an exploratory point of view. We suggest the use of more structured interviews further along the process, where there is a proposed solution or hypothesis that needs to be validated or refuted. We exemplify interviews in the class, go through tips for the field [40] and review potential pitfalls [78].

#### 2.5.5. Qualitative Data Analysis

An initial introduction to qualitative data analysis (QDA) allows students to see the importance of collecting data thoroughly. After collecting visual, audiovisual, and textual data, there needs to be a way to make sense of the information collected. Based on Russell Bernard and Ryan [79], we review two approaches for text analysis: content analysis and grounded theory [80,81]. We use *The Coding Manual for Qualitative Research* by Johnny Saldaña [82] to go over examples of coding textual data. We also discuss the dangers of relying on code frequency as a form of elevating insights. Under a constructivist perspective, we tend to highlight grounded theory as the strategy to create inductive themes and emergent themes from the field notes, interviews, and other sorts of textual raw data. We discuss the importance of iteration, intercoder reliability, negotiated codes, and salient theories. Coding as a form of analysis is a “judgment call” [83]. 

## 3. Results

The following section presents four designs for healthcare projects worked by engineers and biomedical engineers that had undergone the anthro-design training or qualitative research concepts grounded in anthropological theory. When these students were prompted with a challenge or an open-ended question by a clinical mentor, clinical sponsor, or someone in the health area, they had to undergo a qualitative research process to understand the issue and the specific context. While describing the examples, we narrate the way how the process prepped to develop these systems that are far from designing isolated artifacts. 

**Figure 3:**
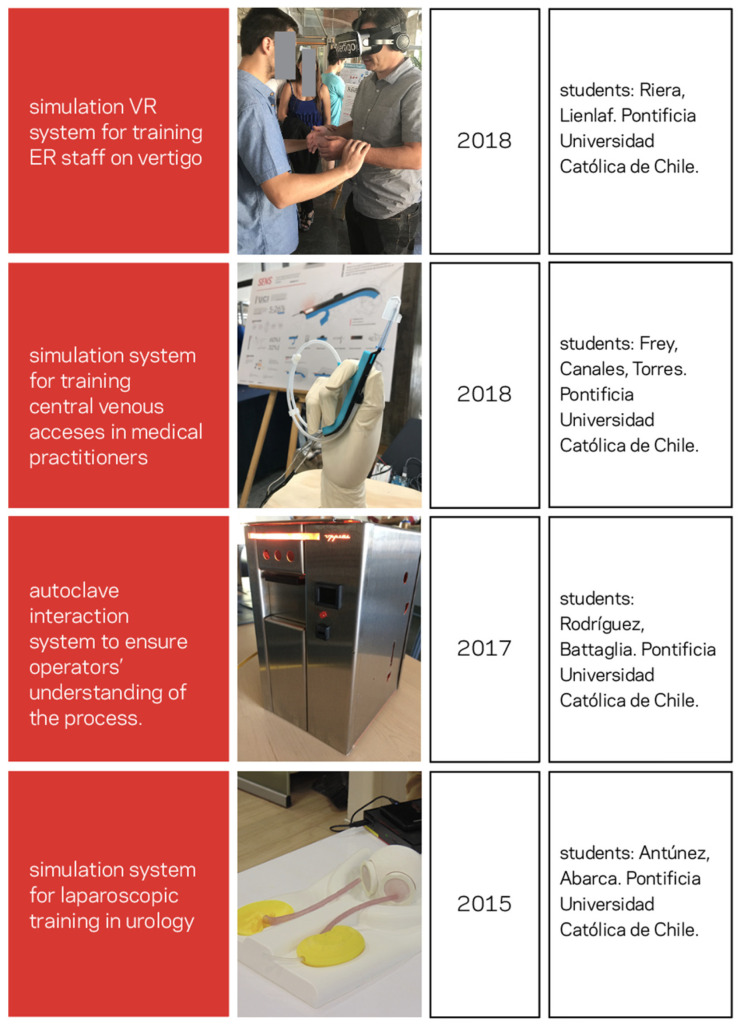
Example of healthcare designs developed by undergraduate students after having been trained in qualitative methods grounded in an anthropological framework.

Figure 3 shows four validated proof of concept prototypes that derive from an applied study of culture and behavior. These correspond to projects undertaken in different years and by different undergraduate students majoring in engineering, design and innovation. We will briefly describe them to examine how the human component is involved in the design. Elements of physiopathology will not be described in detail since that is not the purpose of this paper. 

### 3.1. A Simulation VR System for Training ER and UC Staff on Vertigo

For this pre pandemic project, the clinical sponsor came in with the issue of having underdiagnosed vertigo patients in the ER who could be sent home without being tested for a brain hemorrhage. While carrying out qualitative research in the ER and visiting multiple vertigo patients, students realized that vertigo patients were usually tagged as overreacting nervous people whose vertigo was not taken seriously by some of the staff. This was an at-risk population suffering a power imbalance. Patient information was not taken into consideration because medical professionals could not understand what was happening to them. While researching the context, students realized that there was a lack of medical training in how to empathize with this condition. In a few cases, students realized that faculty members were inducing vertigo to show the medical students that it was a real condition. After carrying out several interviews, shadowing in the ER and UC, and working very closely with medical professionals and patients, the students thought that they had to develop a way to make staff understand what a patient with vertigo was going through, so they were not prone to overlook the condition. The system consisted in an educational instructional component, a VR simulation displayed in an oculus rift headset and a moving base. This would be included in the medical healthcare training as part of their simulation program for the ER rotation. This particular project showed how the involving thorough qualitative research can lead to a solution based on an understanding of the context and clashing perceptions (patient vs. medical personnel). 

### 3.2. Improving the Context of Operation and Communication of an Autoclave

Autoclaves are present in most healthcare environments. They can work for sterilizing instruments in hospitals. Another part of our project concerned issues of improper instrument sterilization in a hospital. After going through a complex process for gaining access to the hospital network, the students spent a long time interviewing and observing autoclave operators and medical administrators. The biggest issue was that when the material was not correctly sterilized, surgeries were delayed or had to be rescheduled. This is the case for many public hospitals that do not have extensive access to medical instruments. Operators did not know when the autoclaving process had completed instrument sterilization. The students also immersed themselves in the culture of autoclave manufacturing and learned that companies were concentrating on competing with other manufacturers rather than on what was happening in the field. Interfaces are a very culturally rooted touchpoint for any design. It has to do with language, perception, and ways people operate in a particular sociotechnical system. These machines produced or adjusted to the cultural understanding of the operators. There was also a power imbalance observed by the students in how these “mistakes” were reported and how the operators were treated when the sterilization process did not work. Using the anthropologically grounded techniques, the students immersed themselves in the operators’ culture to better understand their cognitive load, their behaviors, and how they could better manage the machines. They worked with the manufacturing company to understand the feasibility from a production perspective, gaining their trust by working collaboratively. The new developed systems represent the voices of operators, medical administrators, and the manufacturers. They were designed looking to achieve multivocality [61].

### 3.3. Two Simulation Devices

The last two systems were developed with a difference of three years and by different students who took the anthro-design course. Both groups of students engaged in a semester relationship with their clinical sponsors to develop new technologies in the areas of simulation. The group working in 2018 concentrated on developing better training in central venous accesses (CVA), and the one in 2015 worked to improve training in sutures and laparoscopy for urology. Even though these two groups dealt with two different medical specialties and procedures, both dealt with education, training, and medical procedural tasks. The research in both projects involved interviews, extended periods of observation, understanding the cultural underpinnings of the practice, and qualitative data analysis of all textual and audiovisual data collected during the field research. In these immersions, students created rigorous qualitative research protocols, spent several hours with the clinicians observing the real and observed procedures and conducted interviews with instructors and medical professional. Not only did the students develop systems that were prompted by technology, they also understood the points of view, the details of the practice, the emotional goals for those involved (very in sync with educational goals from the practitioners), and the cultural underpinnings on how medical training is carried out in these institutions.

## 4. Discussion

The benefits of educating biomedical engineers, engineering designers, or others designing in the healthcare environment in thorough qualitative research are clear. As published in the article “Embracing the Social Turn: Epistemic Change in Engineering Students Enrolled in an Anthro-design Course” [21], students who understand the importance of going beyond the user set the basis for a more humane engineering design practice. Students also gain a new understanding of qualitative research and its methodological rigor. In healthcare, this sets the stage for not only understanding the patient, but also to appreciate what other disciplines can bring to the conversation in healthcare technologies and experiences [33]. Disciplines that have been trained under a positivist paradigm can incorporate a more holistic approach in the development of socially responsible technologies [31,84].

This article has shown the outcomes in teaching five concepts of qualitative research grounded in anthropological theory. Using examples from real students having undergone formal anthropological instruction at the undergraduate level, we showed how engineering and biomedical engineers use these concepts to develop technologies for healthcare that are meant to be multivocal and respectful of disparities and cultural systems. Design tactics and heuristics have already been incorporated into biomedical engineering education [85,86,87]. However, few studies have shown the potential of qualitative research training for enhancing biomedical engineering students’ self-awareness, understanding of environments, and observation skills during that process [85,88].

This article is a contribution to the instruction of biomedical engineers, engineer designers, and other design professionals approaching healthcare design. The current methods to approach qualitative research in the initial phases of the design process may lack the depth that qualitative research grounded in humanistic epistemic frameworks might bring. Engaging with open-ended problems is a major component in building interdisciplinary competencies and expertise in the education of future engineers and biomedical engineers. However, this might not be enough. It is our belief that future biomedical engineers or others designing in the health area can profit from a framework and applicable tools from the humanities to face the cultural particularities of the medical field embedded in these open-ended challenges. This could lead to the design of more ethical innovations [89] and more prepared professionals for a more diverse workplace and a more diverse end-user population.

Throughout this work, and adding to prior research, we have shown the value of instructing five core qualitative research concepts to engineer designers in projects regarding healthcare. We argue that the epistemic framework of anthropology and QAME (questions, assumptions, methods, and evidence) as posed by Elizabeth “Dori” Tunstall, [90] and the transdisciplinary value of ethnography can provide the much-needed ethical standing for educating engineer designers that can represent the complexity and multivocality required in healthcare design. Further work involves the assessment of the impact of the use of the techniques in recent years and in other courses.

## 5. Conclusions

In this paper we explore five key concepts from ethnographic research that could help healthcare education better prepare their students for the socio-technical complexities of professional practice and current healthcare trends that emphasize a patient-centered approach. These concepts are informed consent, field notes, observation, open-ended interviews and qualitative data analysis. We assert that these concepts are critical for healthcare programs seeking to move beyond a rigid or not methodologically grounded design-thinking approach to student projects. Through a description of the application of these concepts in real classrooms, we are able to observe their impact in students’ final products. In these projects we were able to explore how qualitative tools led to more socially and psychologically aware results. Ultimately, we believe that these concepts should be regarded as mandatory content for health students navigating the complexities of our socio-technical reality.

## Data Availability

Not applicable.
